# Enhanced Photocatalytic CO_2_ Reduction with Incorporation of WO_3_ Cocatalyst in g-C_3_N_4_-TiO_2_ Heterojunction

**DOI:** 10.3390/molecules30112317

**Published:** 2025-05-25

**Authors:** Yiting Huo, Zhen Wu, Yanhui Yang, Bin Dong, Zhidong Chang

**Affiliations:** 1 School of Chemistry and Biological Engineering, University of Science and Technology Beijing, Beijing 100083, China; hytchem@oit.edu.cn (Y.H.); dongbin@ustb.edu.cn (B.D.); 2 School of Chemical Engineering, Ordos Institute of Technology, Ordos 017010, China; wu9_9@163.com; 3 Ordos Laboratory, Ordos 017010, China; 4 School of Chemistry and Molecular Engineering, Nanjing Tech University, Nanjing 211816, China

**Keywords:** photocatalytic CO_2_ reduction, WO_3_ cocatalyst, g-C_3_N_4_-TiO_2_ heterojunction, oxygen vacancy

## Abstract

To enhance the performance of photocatalytic CO_2_ reduction, the development of suitable cocatalysts represents an effective strategy. Cocatalysts can interact with photocatalysts to improve light absorption capabilities and facilitate the separation and transfer of photogenerated electrons and holes. Moreover, they provide highly active surface sites that promote the adsorption and activation of CO_2_, which leads to acceleration of photocatalytic reduction. Herein, WO_3_ is employed as a cocatalyst to promote the CO_2_ photoreduction performance of a g-C_3_N_4_-TiO_2_ heterojunction through a facile and scalable calcination method. In pure water, optimal WO_3_/g-C_3_N_4_-TiO_2_ (WCT) delivers high selectivity CO and CH_4_ formation of 48.31 µmol·g^−1^ and 77.18 µmol·g^−1^ in the absence of a sacrificial reagent and extra photosensitizer, roughly 13.9 and 45.7 times higher than that of g-C_3_N_4_-TiO_2_ (CT). WO_3_ can strongly interact with g-C_3_N_4_-TiO_2_ electronically, guiding electrons across the interface to the surface. The oxygen vacancies in WO_3_, as electron-enriched centers, not only enhance charge separation and form efficient charge transfer channels but also capture photogenerated electrons to suppress charge recombination. This strong interaction and oxygen vacancies in WO_3_ jointly improve photocatalytic CO_2_ reduction activity and selectivity, offering a feasible way to design efficient cocatalysts.

## 1. Introduction

Utilizing excess CO_2_ as a carbon raw material to generate value-added compounds via catalytic reactions is one of the viable strategies for reducing CO_2_ emissions. Thus, exploring photocatalytic CO_2_ reduction into C1 solar fuels such as CO and CH_4_, which can decrease CO_2_ content in the environment and reduce humanity’s reliance on petroleum and other products, holds great promise in curbing global warming [1,2,3,4]. The predominant limitations of reported photocatalysts for CO_2_ reduction include low efficiency, high cost, and toxicity [5,6]. In particular, slow charge dynamics including insufficient light absorption and high recombination rates of electron–hole pairs, together with high activation energies, severely restrict photocatalytic CO_2_ activity [7,8,9]. Consequently, approaches like band-gap engineering through metal or non-metal element doping, deposition of noble metals, or the incorporation of cocatalysts [10,11] have been proposed to tackle these problems.

The incorporation of cocatalysts into photocatalysts is an effective way to enhance CO_2_ photoreduction performance. Cocatalysts can promote surface charge separation, crucial for efficient charge transfer; boost the quantity of active sites by modifying the photocatalyst’s surface structure and electronics; reduce CO_2_ conversion activation energy, making the reaction more thermodynamically favorable and accelerating product formation; enhance selectivity toward target molecules like CO, methane, or formic acid, and improve the photocatalytic system’s stability by alleviating excess charge accumulation; optimize the reaction pathway, guiding reactants through beneficial steps to lower the energy barrier and minimize side reactions; and some with unique optical properties can enhance light harvesting by absorbing specific wavelengths of light and transferring energy to photocatalysts, expanding the light-response range. This multifaceted role of cocatalysts shows great potential for developing more efficient and sustainable photocatalytic CO_2_ utilization systems [12,13]. Among the reported cocatalysts, tungsten trioxide (WO_3_), as an oxide of transition metal elements, exhibits distinctive properties. Characterized by a relatively wide band-gap energy spanning from 2.4 to 2.8 eV, WO_3_ is capable of absorbing a broader spectrum of visible light, which endows it with enhanced attractiveness [14]. The synergistic effect with other materials can not only improve the light absorption performance and carrier separation efficiency of the photocatalyst but also adjust the surface properties and active site distribution of the catalyst, thereby improving the overall performance of the photocatalyst in CO_2_ adsorption and activation processes, and thereby enhancing catalytic efficiency. Moreover, WO_3_ emerges as a highly prospective candidate for photocatalysts on account of its non-toxicity, physicochemical stability, and remarkable resistance to photo-corrosion [15].

Concurrently, graphite carbon nitride (g-C_3_N_4_), due to its unique band structure and ease of fabrication, has become a prominent material in the field of photocatalysis [16,17,18]. This particular material exhibits a low band gap of 2.7 eV, minimal particle presence, non-toxicity, cost-effectiveness, favorable controllability of the structure, and superior electronic and optical properties [19]. This material also enhances the photocatalytic activity of the composite by extending the absorption band. Nevertheless, the low electrical conductivity, relatively small surface area of g-C_3_N_4_, as well as the rapid recombination of electrons and holes, limit its photocatalytic activity [20]. A promising aspect is that the integration of g-C_3_N_4_ and TiO_2_ in a heterojunction configuration has demonstrated remarkable photocatalytic efficiency [21]. The photoreduction of CO_2_ based on a g-C_3_N_4_/TiO_2_ heterojunction was studied to produce solar fuel. For instance, Wang et al. [22] fabricated g-C_3_N_4_–based composite photocatalysts modified with titanium dioxide (TiO_2_) through ball-milling and calcination for the photoreduction of CO_2_ to methane and carbon monoxide. Zhang et al. [23] constructed 3D/2D TiO_2_/p-g-C_3_N_4_ micro-nano heteroarchitectures through a facile acid hydrothermal route, which improved the performance of photocatalytic CO_2_ reduction. Although the g-C_3_N_4_-TiO_2_ heterostructure has shown good stability in CO_2_ reduction, the technology based on the g-C_3_N_4_-TiO_2_ heterostructure photocatalyst is not mature enough to obtain the ideal product possessing a high yield along with high selectivity.

In this study, the incorporation of a WO_3_ cocatalyst into a g-C_3_N_4_-TiO_2_ heterojunction was attempted to fabricate WO_3_/g-C_3_N_4_-TiO_2_ photocatalysts for enhanced photocatalytic CO_2_ reduction. A detailed synthesis process was elucidated; a comprehensive characterization of the catalysts was performed; and the mechanisms underlying the enhanced photocatalytic activity were revealed. These materials demonstrated excellent high photocatalytic activity and chemical stability for carbon dioxide reduction due to enhanced charge separation and transfer.

## 2. Results and Discussion

### 2.1. Characterizations of WO_3_/g-C_3_N_4_-TiO_2_

**Figure 1:**
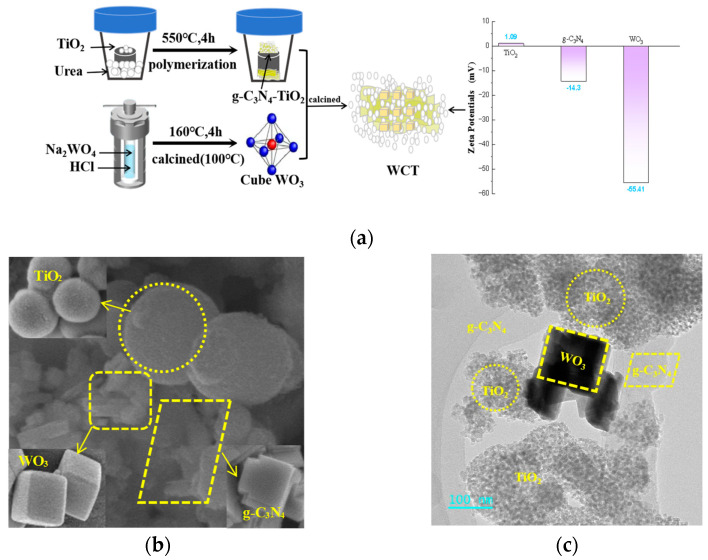

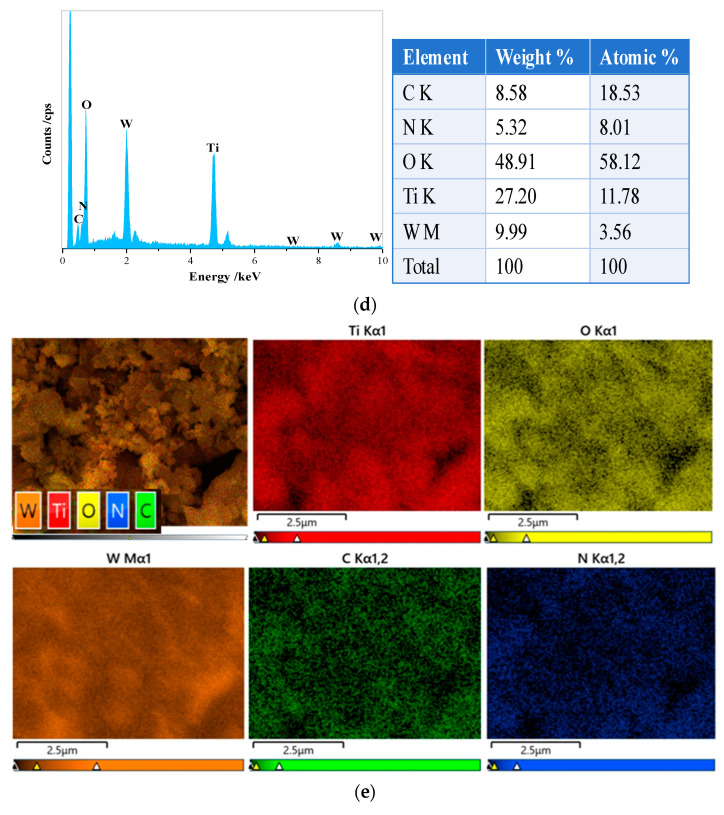
Material preparation process and the Zeta potential of TiO_2_, WO_3_, and g−C_3_N_4_ (**a**); SEM image of WO_3_/g−C_3_N_4_-TiO_2_ and TiO_2_, WO_3_, and g−C_3_N_4_ (**b**); HRTEM image of WO_3_/g−C_3_N_4_−TiO_2_ (**c**); SEM image of WO_3_/g−C_3_N_4_-TiO_2_ with corresponding EDS (**d**); elemental mapping of WO_3_/g−C_3_N_4_−TiO_2_ (**e**).

WO_3_/g-C_3_N_4_-TiO_2_ samples were synthesized by the calcining method as depicted in Figure 1a. The Zeta potential of TiO_2_ is 1.09 mV and that of WO_3_ is −55.41 mV, which favors the formation of the interfaces between TiO_2_ and WO_3_, and consequently, a strong interaction among these semiconductors, along with more light absorption pathways. The morphology of WO_3_/g-C_3_N_4_-TiO_2_ was probed further via SEM, showing a two-dimensional layered structure of g-C_3_N_4_ and a sphere structure of TiO_2_, which are shown in Figure 1b. WO_3_ synthesized by the hydrothermal method has a cubic configuration, and uniformly disperses over TiO_2_. Some g-C_3_N_4_ sheets are also observed to be attached to WO_3_. In this regard, WO_3_/g-C_3_N_4_-TiO_2_ with a good interaction to construct composite is achieved. More interestingly, the HRTEM images in Figure 1c provide further evidence of the strong interaction existing among these three components. The microspheres of TiO_2_ with multiple channels were seen as well as WO_3_ possessing a cubic structure, which facilitates the formation of oxygen vacancies [24]. Furthermore, the combined structure of these components is characterized by enhanced compactness, facilitating the separation of charge carriers and ensuring efficient electron transport. Thus, incorporation of a WO_3_ cocatalyst into the g-C_3_N_4_-TiO_2_ heterojunction resulted in better optical radiation penetration accompanied by an efficient adsorption process to maximize photoactivity. Further characterization of the catalyst’s elemental composition was carried out using energy-dispersive X-ray spectroscopy (EDS). These results presented in Figure 1d reveal that C, N, O, Ti, and W are dispersed throughout the composite photocatalyst. Elemental mapping of WO_3_/g-C_3_N_4_-TiO_2_ further indicates the uniform distribution. Moreover, they demonstrate the successful binding of WO_3_ to the surface of g-C_3_N_4_-TiO_2_. Based on these findings, the WCT heterojunction has been successfully synthesized, which is conducive to efficient light absorption and charge carrier separation.

**Figure 2:**
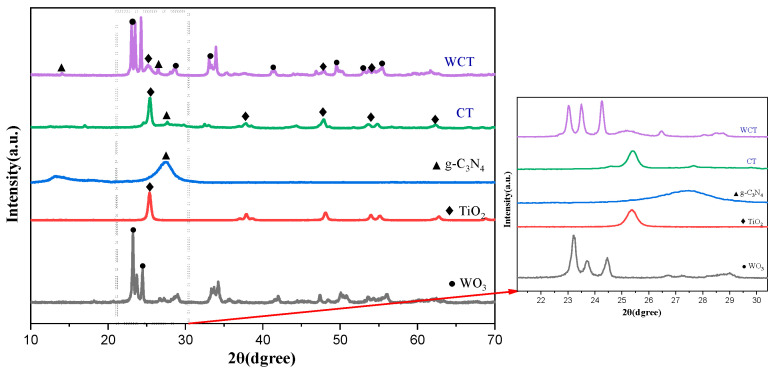
XRD patterns of WO_3_, TiO_2_, g-C_3_N_4_, CT, and WCT.

The results of XRD are displayed in Figure 2 to characterize the phase structure of the prepared photocatalysts. The characteristic peaks of pristine WO_3_ are detected at 2θ of 23.10°, 23.62°, 24.38°, 26.54°, 28.72°, 33.32°, and 34.22°. These peaks are associated with the monoclinic phase of WO_3_ according to the JCPDS, Card No. 83-0951 [25]. For g-C_3_N_4_, the peaks at 13.4° and 27.3° correspond to (100) and (002) crystal planes of g-C_3_N_4_ (JCPDS, Card No.87-1526). In g-C_3_N_4_, the (002) plane is attributed to interplanar stacking of the aromatic system, whereas the (100) plane is associated with the in-plane structural packing motif [26]. The peaks of TiO_2_ match those of the standard PDF card (JCPDS, Card No.21-1272), which provides an indication that the experimental specimens are constituted purely of anatase TiO_2_ [27]. Regarding the WO_3_/g-C_3_N_4_-TiO_2_ composite, characteristic peaks belonging to TiO_2_, g-C_3_N_4_, and WO_3_ are clearly visible. Notably, the characteristic (002) diffraction peak of g-C₃N₄ shifted from 27.3° to 26.5° upon composite formation with WO_3_/g-C_3_N_4_-TiO_2_, demonstrating lattice expansion and interfacial electronic coupling effects [24]. Moreover, no impurity-related peaks are detected in the XRD pattern, confirming the high purity of the as-synthesized samples. This result further demonstrates successful fabrication of the composite without significant distortion of the original crystal structures of the individual components.

**Figure 3:**
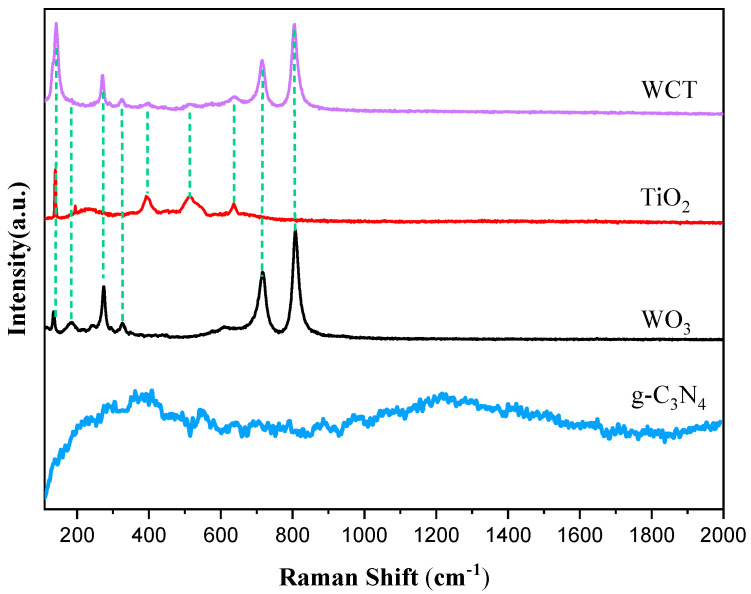
Raman spectra of WO_3_, TiO_2_, g−C_3_N_4_, and WCT.

As depicted in Figure 3, to study the structural features of the samples, Raman spectra were obtained. For pure WO_3_, characteristic bands are clearly discernible at 131.92, 272.46, 716.92, and 807.41 cm^−1^. The peak at 272.46 cm^−1^ is attributable to the bending vibration of the W–O bond, while the peaks at 716.92 and 807.41 cm^−1^ are indicative of the stretching vibration mode of the O–W–O bands. These results are consistent with the previous ones [28,29]. Analogously, the Raman peaks at 360.1 and 1229.6 cm^−1^ belong to the polymer-based structure of g-C_3_N_4_ [30]. The absence of D and G bands was probably accounted for by the relatively low content of g-C₃N₄ revealed by EDS, along with its poor crystallinity indicated by the indistinct XRD peaks [31]. Anatase TiO_2_ primarily consisted of peaks at 143.41, 199.35, 396.22, and 519.64 cm^−1^. Specifically, the peaks at 143.41 and 396.22 cm^−1^ correspond to vibration of the Ti–O bond, while the peak at 199.35 cm^−1^ is attributed to vibration of the Ti–O–Ti bond. The peak at 519.64 cm^−1^ is associated with vibration of the O–Ti–O bond [32,33]. In the distinctive characteristic patterns of the WO_3_/g-C_3_N_4_-TiO_2_ (WCT) composite, Raman modes appear at 142.21, 271.21, 716.17, and 808.95 cm^−1^. It is possible that due to the low content and poor crystallinity of g-C_3_N_4_, as well as it being encapsulated, the peaks of graphitic carbon nitride were not observed in the WCT composite. A slight shift was observed in the peaks of WO_3_ and TiO_2_, indicating an interaction between WO_3_ and TiO_2_ that results in the formation of a composite facilitating efficient charge carrier transfer. Furthermore, the distinct vibrational peak of WO_3_ in the Raman spectrum of the composite indicates that WO_3_ within the WCT composite possesses remarkable crystallinity.

### 2.2. CO_2_ Photoreduction Efficiencies

**Figure 4:**
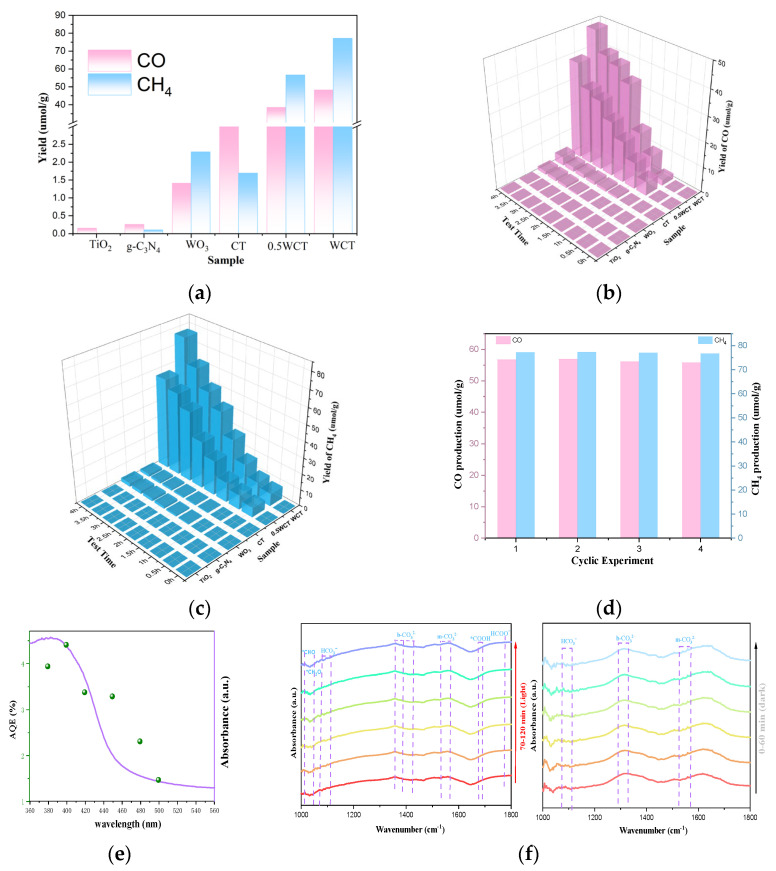
(**a**) Yields of CO and CH_4_; (**b**) photocatalytic activity of CO; (**c**) photocatalytic activity of CH_4_; (**d**) the cyclic experiment results of WCT; (**e**) wavelength−dependent AQE and the UV−vis absorption spectrum of WCT; (**f**) in situ DRIFT spectra for photocatalytic CO_2_ reduction over WCT.

To assess the effectiveness and catalytic activity of the WO_3_/g-C_3_N_4_-TiO_2_ photocatalyst, the CO_2_ reduction performance of the prepared materials was assessed under xenon lamp illumination in water without additional organic sacrificial agents (such as triethanolamine) or photosensitizers. And a series of conditional blank control experiments were carried out; the data can be found in Table S1 of the Supplementary File. The main reduction products were identified as CO and CH_4_, and nothing was detected in the dark or in the absence of photocatalysts or CO_2_. The evolution of CO and CH_4_ for all samples was observed in (Figure 4). The WO_3_/g-C_3_N_4_-TiO_2_ (WCT) samples exhibited boosted photocatalytic performance in reducing of CO_2_ to solar fuel C1 products, among which WCT displays a maximum CO production yield of 48.31 µmol·g^−1^and a maximum CH_4_ production yield of 77.18 µmol·g^−1^, roughly 13.9 and 45.7 times higher than that of CT (3.53 and 1.70 µmol·g^−1^, respectively). Furthermore, with increased loading of WO_3_, the photocatalytic performance of WCT is markedly enhanced. From Figure 4b,c, the yield of CO and CH_4_ for WCT samples are 1.25 and 1.36 times higher than 0.5 WCT samples. After carrying out the reaction for a four-cycle experiment, the activity decrease is negligible, revealing the good photocatalytic stability of WCT (Figure 4d). The apparent quantum efficiency (AQE) of CO_2_ photoreduction was measured at different monochromatic wavelengths including 380, 400, 420, 450, 480, and 500 nm over the WCT sample. Clearly, the trend of AQE matches well with the light absorption spectrum of WCT (Figure 4e) and the value of AQE reaches 4.41% at a 400 nm wavelength (Table S4 in Supplementary File), affirming the photocatalytic nature of CO_2_ reduction. In order to uncover the underlying reasons for the high production yield of CH_4_, in situ diffuse reflectance infrared Fourier-transform spectroscopy (DRIFTS) was explored. When CO_2_/H_2_O is introduced into the system in the dark, the chemisorption of CO_2_ on the catalyst surface is detected, evidenced by the appearance of peaks corresponding to HCO_3_^−^, m-CO_3_^2−^, and b-CO_3_^2−^ species [34]. Under light irradiation, adsorption peaks associated with *COOH (at 1669 cm^−1^), *CHO (at 1012 cm^−1^), and *CH_3_O (at 1042 cm^−1^) emerge. These species are crucial intermediates in the process of converting CO_2_ into CH_4_.

Additionally, a comparison of the photocatalytic performance between the WCT samples and other previously reported advanced photocatalysts in the literature is summarized in Table 1.

### 2.3. Insight into Increased Photocatalytic Activity

**Figure 5:**
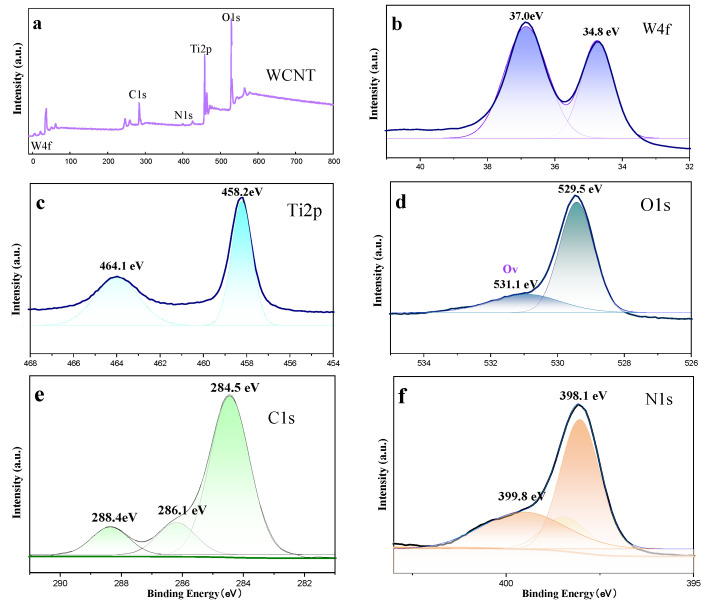
The XPS survey spectra of the WCT sample (**a**) and XPS of W4f (**b**), Ti2p (**c**), O1s (**d**), C1s (**e**), and N1s (**f**) of the WCT sample.

Figure 5a presents the XPS scan spectrum of WCT. As can be observed from this figure, WCT encompasses signals of the elements of tungsten, carbon, nitrogen, titanium, and oxygen. The signal peaks of these elements can be deconvoluted to conduct a more in-depth analysis of their chemical states. The high resolution W4f spectrum in Figure 5b shows binding energies of 34.8 and 37.0 eV for W4f_7/2_ and W4f_5/2_, respectively, corresponding to the W^+6^ oxidation state of WO_3_ [38]. From Figure 5c, the signals of Ti2P_3/2_ and Ti2P_1/2_ in TiO_2_ are located at the peaks of Ti2p at 458.2 and 464.1 eV. Figure 5d depicts the XPS spectrum of O1s with two peaks at 529.5 and 531.1 eV. The strong O1s peak at 529.5 eV is ascribed to the lattice O atom in the WCT composite. Owing to WO_3_ lattice O-defects and surface-adsorbed OH^−^/O_2_^−^ [39], the intensity of peak at binding energy 531.1 eV decreases. Regarding Figure 5e, it reveals the C1s orbital spectrum of WCT, featuring three characteristic peaks at binding energies of 284.5 eV, 286.1 eV, and 288.4 eV. These peaks are each assigned to the sp^2^ C–C, C–N, and N–C=N bonds in g-C_3_N_4_. The N1s spectrum in Figure 5f, t can be split into peaks at 398.1 eV and 399.8 eV, which respectively correspond to the C=N–C and N–C bonds in g-C_3_N_4_ [40]. In conclusion, the successful synthesis of the WO_3_/g-C_3_N_4_-TiO_2_ (WCT) composites is further supported by X-ray photoelectron spectroscopy (XPS) analysis of the composite specimens. This analysis also provides additional evidence that aligns with the results obtained from other characterization methods.

**Figure 6:**
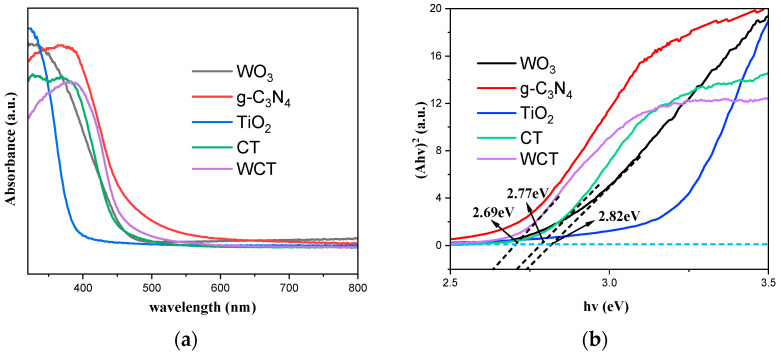
(**a**) The spectra acquired from UV–vis DRS; (**b**) the plot of (αhν)^2^ versus the energy (hν) of the samples.

To investigate light-harvesting capabilities and the band gap of the samples, UV–vis DRS spectra were obtained. Figure 6a depicts the UV–vis DRS spectra of TiO_2_, g-C_3_N_4_, WO_3_, CT, and WCT samples. The incorporation of WO_3_ significantly enhances the light absorption capacity of g-C_3_N_4_-TiO_2_ (CT), thereby exerting a considerable effect on the photocatalytic process. Moreover, narrower band-gap energies (Egs) can enhance the photoelectric conversion efficiency of composite materials, which is crucial for the photocatalytic process. The band gaps were determined by Tauc plots in Figure 6b. The band-gap energies (Egs) of pure WCT, CT, and WO_3_ were calculated to be 2.69, 2.77, and 2.81 eV, respectively [41]. This can be attributable to WO_3_–induced mid-gap states (XPS in Figure 5) and interfacial charge transfer. This enhanced light harvesting directly correlates with a 36% improvement in CH_4_ production (Figure 4c), confirming the critical role of band engineering.

**Figure 7:**
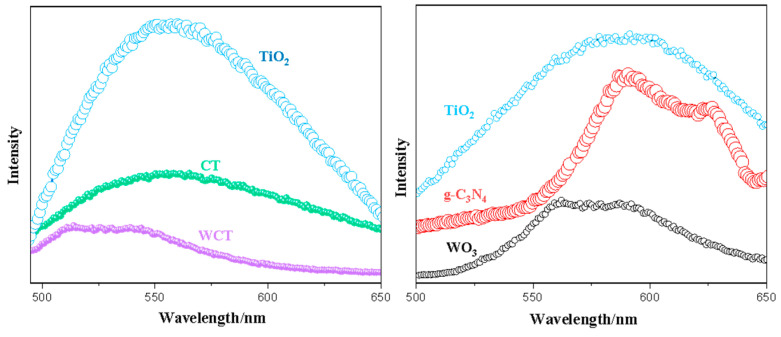
PL analysis of TiO_2_, g-C_3_N_4_, WO_3_, CT, and WCT samples.

Photocatalytic activity depends mainly on efficient charge separation and transport. A higher photoluminescence (PL) intensity indicates a lower charge separation efficiency and leads to a higher electron–hole recombination rate. Photoluminescence carrier production and its recombination can be evaluated by photoluminescence (PL) analysis. Figure 7 shows the charge separation efficiency of the TiO_2_, g-C_3_N_4_, WO_3_, CT, and WCT composite samples. Using pristine TiO_2_, a strong emission peak appears, owning to faster recombination of photogenerated electron–hole pairs on the catalyst surface [42]. When coupling TiO_2_ with g-C_3_N_4_, the CT sample’s PL intensity experiences a gradual yet significant decrease, which is attributed to superior charge carrier separation [43,44]. Moreover, the incorporation of WO_3_ in a g-C_3_N_4_-TiO_2_ heterojunction to construct the WCT composite is found to be efficient for the separation and transfer of electrons; analogous discoveries have been presented in the relevant literature [45]. Consequently, the construction of a WO_3_/g-C_3_N_4_-TiO_2_ heterojunction can facilitate the efficient generation and separation of charge carriers, which is advantageous for the effective reduction of CO_2_ under visible light.

**Figure 8:**
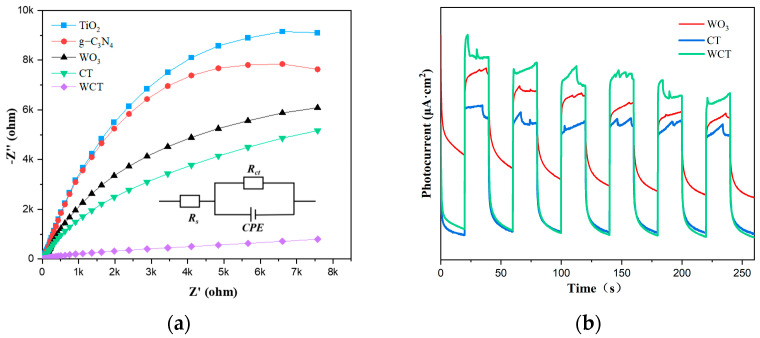
(**a**) Electrochemical impedance spectroscopy and (**b**) the transient photocurrent response of the prepared photocatalysts.

To gain deep insights into the electron excitation processes, charge migration mechanisms, and kinetic characteristics such as charge transfer rates of the as-prepared catalysts, measurements of photocurrent responses and analyses of electrochemical impedance spectroscopy (EIS) were carried out. A Nyquist plot of TiO_2_, WO_3_, g-C_3_N_4_, CT, and WCT is depicted in Figure 8a. In electrochemical impedance spectroscopy (EIS), there is generally a positive correlation between the arc diameter in the Nyquist plot and the electron transport resistance exhibited by the catalyst. The arc radius corresponding to the WCT–based electrode is smaller in comparison to TiO_2_, g-C_3_N_4_, and CT, resulting from the incorporation of WO_3_ into g-C_3_N_4_-TiO_2_. Therefore, due to the higher photocatalytic activity of WCT compared to CT, this signifies the lowest charge transfer resistance (Rct) among WCT catalysts. This facilitates the separation of charge carriers and enables the rapid separation of photogenerated electron–hole pairs [46,47]. Moreover, the electronic interactions of the prepared photocatalysts were studied through photocurrent measurements using an electrochemical workstation. As depicted in Figure 8b, the chronoamperometric current–time (I–t) curve is presented. The photocurrent measurements were obtained under multiple light-on and light-off conditions. In comparison to CT and WO_3_, the photocurrent density of WCT is the highest, manifesting the highest separation efficiency of carriers for WCT. Consistent with the PL results is the fact that the photocurrent response of WCT is higher than that of CT and pure WO_3_. The WCT composites exhibit a significantly higher photocurrent, providing compelling evidence of enhanced charge separation efficiency (e^−^-h^+^). This improvement enables the generation of a greater number of photogenerated electrons, thereby substantially augmenting photocatalytic activity. The aforementioned results unequivocally demonstrate that the integration of WO_3_ into the g-C_3_N_4_-TiO_2_ heterojunction is conducive to promoting photocatalytic performance by facilitating the separation and transfer of carriers.

**Figure 9:**
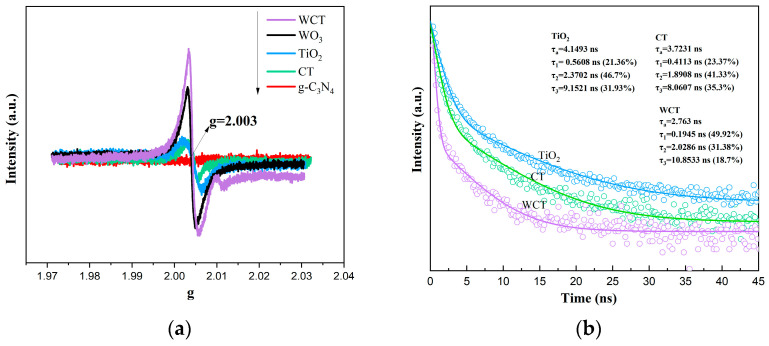
EPR spectra of samples (**a**); TRPL spectra of TiO_2_, CT, and WCT (**b**).

Oxygen vacancies (Ovs), as intrinsic defects, can modulate the local geometric structure of metal oxides, thereby tailoring their physical and chemical properties [48]. This modulation influences the band alignment of metal oxides and introduces defect levels, which significantly enhances light absorption. Additionally, the existence of Ovs alters the charge separation and transfer processes within photocatalysts [49]. To investigate the unpaired electrons in WO_3_ with oxygen vacancies, electron paramagnetic resonance (EPR) measurements were conducted. As can be seen from Figure 9a, the signals of different samples (WCT, WO_3_, TiO_2_, CT, and g-C_3_N_4_) exhibit varying intensity around g = 2.003, which corresponds to unpaired electrons located at the Ovs sites, indicating differences in their unpaired-electron environments, likely related to distinct electronic structures or surface properties. It is evident that after the calcination process, the signal intensity at g = 2.003 of WCT markedly increases, confirming the substantial introduction of Ovs into WO_3_ through calcination. The trap states induced by Ovs effectively promote charge separation and facilitate rapid charge transport channels at the heterointerface. Moreover, Ovs enhance light absorption, leading to an increase in photogenerated carriers.

Furthermore, time-resolved photoluminescence (TRPL) spectra can be utilized to explore the dynamic photogenerated charge transfer process. This approach provides more in-depth information, which is beneficial for further validating the underlying mechanism. The TRPL spectra clearly demonstrate that WO_3_/g-C_3_N_4_-TiO_2_ enables ultrafast electron extraction (τ_1_ = 0.19 ns) and promotes long-lived charge separation (with the contribution of τ_3_ reduced to 18.7%). The presence of oxygen vacancies can change the charge distribution on a material’s surface, which in turn affects the separation of electron–hole pairs. On one hand, these oxygen vacancies act as electron-trapping centers, capturing photogenerated electrons from the area near the holes. This process increases the distance between the electrons and holes, reducing their recombination and promoting long-lived charge separation. On the other hand, oxygen vacancies can create a specific electric field distribution on the material’s surface. This electric field helps drive the electrons and holes to move in opposite directions, further enhancing the efficiency of charge separation. Consequently, this reduces the contribution of τ_3_ to 18.7% and supports the achievement of sustained charge separation. These features are crucial for CO_2_ reduction processes, where multi-electron transfer often limits the reaction rate. The 58% reduction in τ compared to TiO_2_ implies that WO_3_ not only effectively suppresses electron–hole recombination but also efficiently channels electrons to the active sites for CO_2_ adsorption and protonation, which are key steps in the formation of CH_4_ or CO [49].

**Figure 10:**
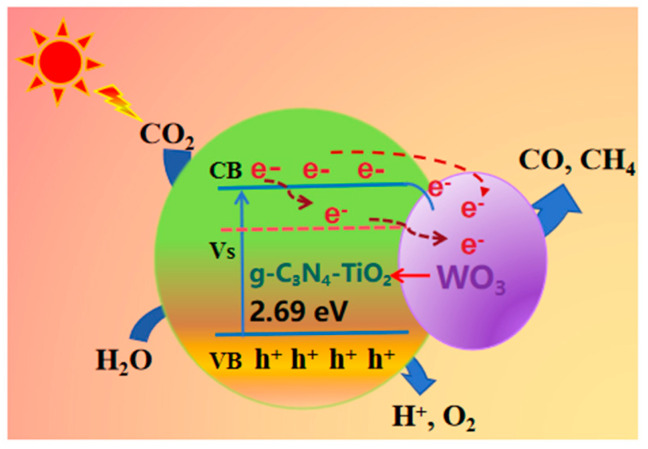
Possible schematics of the mechanisms for charge transfer and photocatalysis.

Based on the above research, we propose the following possible mechanisms in Figure 10: under visible light irradiation in a WO_3_/g-C₃N_4_-TiO_2_ (WCT) composite catalyst, photogenerated electrons from WO_3_ are effectively separated from holes in the TiO_2_ valence band (VB), while electrons from the TiO_2_ conduction band (CB) are separated from holes in g-C_3_N_4_ VB, resulting in increased charge carrier separation efficiency. The incorporation of a WO_3_ cocatalyst into g-C_3_N_4_-TiO_2_ reduces recombination rates, leading to enhanced production of hydroxyl radicals, thereby improving the overall photocatalytic activity of the prepared ternary composite catalysts. The optimized photocatalyst demonstrates efficient performance under visible light illumination with a prolonged lifetime suitable for industrial applications as well.

## 3. Materials and Methods

### 3.1. Materials

Sodium tungstate hydrate, hydrochloric acid, anhydrous ethanol, urea, anatase titanium dioxide, and other chemicals were purchased from Shanghai Maklin Biochemical (Shanghai, China). The experimental water used in this study was deionized water sourced from the laboratory facilities.

### 3.2. Material Preparation

The g-C_3_N_4_-TiO_2_ sample was fabricated by a well-documented one-step vapor deposition method in the presence of urea and anatase titanium dioxide [50]; WO_3_ was obtained through a hydrothermal process [51]. The WO_3_/g-C_3_N_4_-TiO_2_ samples were prepared via a facile calcination method, in particular, as-synthesized WO_3_ and g-C_3_N_4_-TiO_2_ were put into an alumina crucible at a mole ratio of (0.1–1):1 to modulate the quantity of WO_3_, which was subsequently placed in a vacuum tube furnace, calcined for 4 h, cooled to room temperature, and ground into fine powders. The resultant products were denoted as (0.1–1) WCT composite photocatalysts.

### 3.3. Characterization

Characterization methods including powder X-ray diffraction (XRD), Fourier-transform infrared spectroscopy (FTIR), Raman measurements, scanning electron microscopy (SEM) images, a zeta sizer, high-resolution transmission electron microscopy (HRTEM), UV–vis diffuse reflectance spectroscopy (DRS), X-ray photoelectron spectroscopy (XPS), electrochemical impedance spectra (EIS), transient photocurrent responses, electron paramagnetic resonance (EPR), time-resolved photoluminescence (TRPL) spectra, and in situ diffuse reflectance infrared Fourier-transform spectroscopy (DRIFTS) were employed. Information about the instruments used for these tests is provided in the Supplementary File.

### 3.4. Photoreduction of CO_2_

Photocatalytic CO_2_ reduction was investigated using an automatic online trace gas analysis apparatus (Labsolar-6A, Beijing Perfect Light, Beijing, China). Irradiation was carried out using a 300 W xenon lamp equipped with an AM 1.5 filter and operating at a current of 20 A, positioned 2 cm away from the reactor. The gaseous products were then introduced into a gas chromatograph (GC, GC-9790II, Fuli Instruments, Wenling, China; with argon as the carrier gas and a methanizer attached) for detection. Carbon-containing products, including methane, carbon monoxide, methanol, and multi-carbon compounds, were analyzed by a flame ionization detector (FID), while hydrogen, oxygen, and nitrogen were quantified using thermal conductivity detection (TCD). Error bars indicated the standard deviation derived from three independent measurements conducted with 10 mg of fresh sample for each trial.

## 4. Conclusions

In summary, through a facile and scalable calcination method, we have successfully synthesized a WO_3_/g-C_3_N_4_-TiO_2_ nanohybrid characterized by a unique 3D/2D-3D hierarchical heterostructure, accompanied by the presence of oxygen vacancies. A comprehensive suite of advanced characterization techniques, including UV-DRS, PL, EIS, and EPR, was employed to elucidate the underlying mechanisms. The band-gap energy (Eg) of pure WCT was calculated to be 2.69 eV; compared to TiO_2_, g-C_3_N_4_, and CT, the arc radius corresponding to the WCT–based electrode is smaller; the photocurrent density of WCT is the highest; and the signal intensity at g = 2.003 of WCT corresponds to unpaired electrons located at the oxygen vacancy (Ovs) sites. The photocatalytic performance of the WO_3_/g-C_3_N_4_-TiO_2_ (WCT) composite for CO_2_ reduction was evaluated under simulated solar light irradiation without any external photosensitizer or sacrificial agent. Impressively, the WCT composite exhibited outstanding CO_2_ photoreduction activity, yielding CO and CH_4_ with formation rates reaching up to 48.31 µmol·g^−1^ and 77.18 µmol·g^−1^, respectively. These values represent a remarkable improvement over previously reported g-C_3_N_4_-TiO_2_–based photocatalysts for CO_2_ reduction photocatalysts. The presence of oxygen vacancies plays a pivotal role in this process. Oxygen vacancies in WCT act as active sites that enhance the adsorption and activation of CO_2_ molecules, which facilitate the electron transfer processes to enable more efficient conversion of CO_2_ to intermediates. Specifically, the unpaired electrons around oxygen vacancies interact with CO_2_ molecules, weakening C–O bonds and thus promoting the formation of *COOH. Subsequently, the reaction pathways leading to *CHO and *CH_3_O are also accelerated due to the modified electronic environment created by oxygen vacancies. Thus, the reaction rate is significantly increased, while selectivity for CH_4_ is simultaneously improved, attributed to the essential role of oxygen vacancies in the CO_2_ photoreduction process. This research opens up novel prospects for the rational design of other high-efficiency cocatalysts aiming to enhance CO_2_ photoreduction performance.

## Figures and Tables

**Table 1:** The photocatalytic activities of other previous advanced photocatalysts reported in the literature are summarized [34,35,36,37].

Catalyst	CO Yield (μmol·g^–1^·h^–1^)	CH_4_ Yield (μmol·g^–1^·h^–1^)	Fold Increase in CH_4_ Production	AQE (%)	Ref.
Our work: WCT	12.08	19.30	>45	4.41	This study
WO_3_/ZnIn_2_S_4_	44.1	0	0	3.63	[34]
Ru@H-MoO_3-x_	28.0	111.6	8.8	1.05	[35]
strained In_2_S_3_	99.25	11.0	2.11	0.45	[36]
(GeH)_1–x_Si_x_(OH)_0.5_H_x–0.5_	6.91	0	0	5.95	[37]

## Data Availability

The data will be available upon request.

## Supplementary Materials

The following supporting information can be downloaded at https://www.mdpi.com/article/10.3390/molecules30112317/s1, Characterization methods information; the data of a series of conditional blank control experiments; production yield of CO and CH_4_, and the calculated AQE data.

## Abbreviations

The following abbreviations are used in this manuscript:

CT	g-C_3_N_4_-TiO_2_
WCT	WO_3_/g-C_3_N_4_-TiO_2_
