# Supplementary material for: Enhanced Photocatalytic CO2 Reduction with Incorporation of WO3 Cocatalyst in g-C3N4-TiO2 Heterojunction
Source: Molecules. 2025 May 25;30(11):2317. doi: 10.3390/molecules30112317 (PMC12156205; doi:10.3390/molecules30112317)
Supplement: Supplementary file 1 [file molecules-30-02317-s001.zip › molecules-3625353-supplementary.pdf]

data

Data Descriptor

Enhanced photocatalytic CO_2 reduction with incorporation of WO_3 cocatalyst in g-C_3N_4-TiO_2 heterojunction

Yiting Huo[1,2], Zhen Wu[2], Yanhui Yang[3,4*], Bin Dong[1], Zhidong Chang[1,*]

[1] Chemistry and Biological Engineering, University of Science and Technology Beijing, Beijing, China; hytchem@oit.edu.cn, dongbin@ustb.edu.cn, zdchang@ustb.edu.cn
[2] Chemical Engineering, Ordos Institute of Technology, Ordos, China; hytchem@oit.edu.cn, wu9_9@163.com
[3] Ordos Laboratory, Ordos, China; yhyang@njtech.edu.cn
[4] School of Chemistry and Molecular Engineering, Nanjing Tech University, Nanjing, 211816, China; yhyang@njtech.edu.cn
* Correspondence: zdchang@ustb.edu.cn(Z.C); yhyang@njtech.edu.cn(Y.Y)

Abstract: To enhance the performance of photocatalytic CO_2 reduction, the development of suitable cocatalysts represents an effective strategy. Cocatalysts can interact with photocatalysts to improve light absorption capabilities and facilitate the separation and transfer of photogenerated electrons and holes. Moreover, they provide highly active surface sites that promote the adsorption and activation of CO_2, which leads to the acceleration of the photocatalytic reduction. Herein, WO_3 is employed as cocatalyst to promote the CO_2 photoreduction performance of g-C_3N_4-TiO_2 heterojunction through a facile and scalable calcination method. In pure water, the optimal WO_3/g-C_3N_4-TiO_2(WCNT) delivers a high selectivity CO and CH_4 formation of 48.31 μmol·$g^{-1}$and 77.18 μmol·g^{-1} in the absence of sacrificial reagent and extra photosensitizer, roughly 13.9 and 45.7 times higher than that of g-C_3N_4-TiO_2(CNT). WO_3 can strongly interact with g-C_3N_4-TiO_2 electronically, guiding electrons across the interface to the surface.The oxygen vacancies in WO_3, as electron-enriched centers, not only enhance charge separation and form efficient charge-transfer channels but also capture photogenerated electrons to suppress charge recombination.This strong interaction and oxygen vacancies in WO_3 jointly improve the photocatalytic CO_2 reduction activity and selectivity, offering a feasible way to design efficient cocatalysts.

Dataset: DOI number or link to the deposited dataset in cases where the dataset is published or set to be published separately. If the dataset is submitted and will be published as a supplement to this paper in the journal Data, this field will be filled by the editors of the journal. In this case, please make sure to submit the dataset as a supplement when entering your manuscript into our manuscript editorial system.

Dataset License: license under which the dataset is made available (CC0, CC-BY, CC-BY-SA, CC-BY-NC, etc.)

Keywords: photocatalytic CO_2 reduction; WO_3 cocatalyst; g-C_3N_4-TiO_2 heterojunction; oxygen vacancy

3.3 Characterization

Results of Powder X-ray diffraction (XRD) were obtained from a Bruker D2 Phaser diffractometer (Germany) equipped with Cu-Kα radiation. Fourier transform infrared spectroscopy (FTIR) measurements were carried out on a Perkin-Elmer spectrometer employing KBr pellets. The Raman measurements were carried out using a LabRAM HR Evolution UV-VIS-NIR (HORIBA France SAS). Scanning electron microscopy (SEM) images were obtained utilizing a TESCAN MIRA LMS electron microscope. A zeta sizer (Malvern Panalytical ZS90, UK) measured Zeta potential. High-resolution transmission electron microscopy (HRTEM) analyses were carried out using a JEOL JEM-F200 TEM. The acquisition of UV-vis diffuse reflectance spectroscopy (DRS) data was achieved through a Hitachi U-3900 spectrophotometer furnished with an integrating sphere. X-ray photoelectron spectroscopy (XPS) analysis was performed using a Thermo Scientific K-Alpha photoelectron spectrometer. The electrochemical impedance spectra (EIS) and transient photocurrent response were measured at 0.5 V using an Autolab RST5000 electrochemical workstation in a three-electrode setup (Ag/AgCl as standard, Pt as counter, and catalyst-coated FTO glass as working electrode). The electrolyte was 0.1 M Na_2SO_4. The working electrode was prepared by adding 2 mg of photocatalyst to 2 mL of ethanol with 10 μL Nafion, dropping the mixture onto a 1×2 cm FTO glass, and drying at 100 °C for 10 h. Bruker EMXplus provided excellent quality performance for the Electron Paramagnetic Resonance (EPR) research. Time-resolved photoluminescence (TRPL) spectra were collected on a fluorescence lifetime spectrophotometer (FLS 1000, Edinburgh, UK) with an excitation wavelength of 375 nm. In-situ diffuse reflectance infrared Fourier transform spectroscopy (DRIFTS) were acquired on the Nicolet iS50 spectrometer (Thermo Scientific, USA).

Table S1. Blank experiment data

**Condition**	**CO production yield (μmol)**	**CH_4 production yield (μmol)**
Without catalyst	0	0
Without CO_2	0	0
Without H_2O	0.01	0
Without light	0	0
CO_2+H_2O+catalyst+light	0.06763	0

Table S2. CO production yield （μmol/g）

Photocata-lystTime	TiO_2	g-C_3N_4	WO_3	CT	0.5WCT	WCT
0h	0	0	0	0	0	0
0.5h	0.02453	0.09026	0	0	0	0
1h	0.05734	0.11039	0	0	5.56969	2.26662
1.5h	0.09144	0.15328	0	0.72612	10.53398	9.37407
2h	0.10437	0.18276	0.20134	1.01025	16.39427	16.32062
2.5h	0.11895	0.22334	0.53215	1.26733	21.89131	33.33213
3h	0.12764	0.23992	0.96542	1.73637	27.87267	38.14028
3.5h	0.13148	0.24774	1.29535	1.94158	29.95525	40.24708
4h	0.15227	0.25945	1.40977	3.53232	38.52774	48.31102

Table S3. CH_4 production yield （μmol/g）

Photocatalyst Time	TiO_2	g-C_3N_4	WO_3	CT	0.5WCT	WCT
0h	0	0	0	0	0	0
0.5h	0	0.01385	0	0	6.04637	7.90674
1h	0	0.02009	0	0	8.99758	10.43567
1.5h	0	0.03268	0.61025	0.48795	12.28133	17.43565
2h	0	0.04821	0.96733	0.67889	21.63809	28.91376
2.5h	0	0.06145	1.43637	0.85164	29.39459	42.50998
3h	0	0.07014	1.74158	1.16684	44.23351	50.99379
3.5h	0	0.08835	1.95323	1.30474	51.7551	62.0344
4h	0	0.1028	2.28764	1.69526	56.6732	77.18164

The apparent quantum efficiency (AQE) of the products over WCT was calculated as follows:

$$\text{AQE (\%)} = \frac{\text{number of reacted electrons}}{\text{number of incident photons}} \times 100$$

$$= \frac{\text{number of CO molecules} \times 2 + \text{number of CH}_4 \text{ molecules} \times 8}{\text{number of incident photons}} \times 100$$

$$= \frac{(2 \times R_{CO} + 8 \times R_{CH_4}) \times t_1 \times N_A}{p \times t_2 \times \frac{\lambda}{hc}} \times 100$$

where R_{CO} and R_{CH_4} are the CO and CH_4 production rate (mol•h^{-1}); t_1 is the irradiation time (1 h); N_A is Avogadro constant (6.02×1023 mol^{-1}); P is the total incident light flux (W, J•s^{-1}) and equals light intensity per unit area (E, W•cm^{-2}) times effective irradiation area (S, cm^2), where E can be measured by the radiant power energy meter (UV-A and FZ-A, Photoelectric Instrument Factory of Beijing Normal University), S is 4.5 cm^2 in this S5 experiment; t_2 equals 3600 s; λ is the monochromatic light wavelength(m); h is the Planck constant (6.626 × 10^{-34} J•s) and c is the light speed in vacuum (3×108 m•s^{-1}).

Table S4. The calculated AQE at different monochromatic wavelengths over the WCT sample.

λ (nm)	380	400	420	450	480	500
**E (mW/cm²)**	14	15	14	17	20	12
**M_{CO} (ppm)**	327	358	419	369	352	0
**R_{CO} (μmol/h)**	0.11	0.12	0.14	0.13	0.12	0.00
**M_{CH_4} (ppm)**	489	547	546	391	438	221
**R_{CH_4} (μmol/h)**	0.17	0.19	0.19	0.13	0.15	0.07
**AQE (%)**	3.94	4.41	3.38	3.29	2.31	1.47

Disclaimer/Publisher's Note: The statements, opinions and data contained in all publications are solely those of the individual author(s) and contributor(s) and not of MDPI and/or the editor(s). MDPI and/or the editor(s) disclaim responsibility for any injury to people or property resulting from any ideas, methods, instructions or products referred to in the content.
